# Corneal Aberrations and Thickness in Adults Born Small, Appropriate, or Large for Gestational Age at Term

**DOI:** 10.3390/jcm11236903

**Published:** 2022-11-23

**Authors:** Achim Fieß, Jana C. Riedl, Sandra Gißler, Eva Mildenberger, Michael S. Urschitz, Bernhard Stoffelns, Norbert Pfeiffer, Alexander K. Schuster

**Affiliations:** 1 Department of Ophthalmology, University Medical Center of the Johannes Gutenberg University Mainz, 55131 Mainz, Germany; 2 Division of Neonatology, Department of Pediatrics, University Medical Center of the Johannes Gutenberg University Mainz, 55131 Mainz, Germany; 3 Division of Pediatric Epidemiology, Institute for Medical Biostatistics, Epidemiology and Informatics, University Medical Center of the Johannes Gutenberg University Mainz, 55131 Mainz, Germany

**Keywords:** birth weight, small for gestational age, large for gestational age, corneal aberrations, corneal thickness

## Abstract

**Highlights:**

**What are the main findings?**

- Restricted prenatal growth is associated with increased higher-order aberrations in adulthood.
- There was an association between increased higher-order aberrations correlating with lower visual acuity and spherical equivalent.

**What is the implication of the main finding?**

- These results indicate that corneal development is influenced by fetal growth irrespective of prematurity, leading to life-long alterations of ocular shape.
- Increased corneal aberrations caused by adverse fetal growth might be a risk factor for reduced optical image quality in later life.

**Abstract:**

Background/Aims: This study investigated whether there are changes in corneal surface regularity and corneal thickness in adults born small, appropriate, or large for gestational age at term. Methods: This retrospective cohort study involved prospective Scheimpflug imaging of the cornea (Pentacam^®^) to compare the corneal thickness and aberrations between adults classified as small for gestational age (SGA), normal birth weight (BW), and large for gestational age (LGA). Multivariable linear regression was applied to analyze associations with gestational age, BW percentile, placental insufficiency, preeclampsia, and breastfeeding. Results: In total, 448 eyes of 261 individuals born full term (aged 29.9 ± 9.5 years, 140 females) were examined, including 29 severe SGA (BW < 3rd percentile), 32 moderate SGA (BW between 3rd and <10th percentile), 132 normal BW (BW between 10th and 90th percentile), 35 moderate LGA (BW between >90th and 97th percentile), and 33 severe LGA (BW > 97th percentile). There were no differences between groups in the corneal aberrations of the total cornea as well as of the corneal front surface, except for higher-order aberrations in the front of the cornea (*p* = 0.032). There was an association between the increased total root mean square of higher-order aberrations and lower birth weight percentile (*p* = 0.004), with increased higher-order aberrations correlating with lower visual acuity and spherical equivalent. Conclusion: Restricted prenatal growth is associated with increased higher-order aberrations in adulthood.

## 1. Introduction

A low birth weight due to preterm birth influences corneal geometry in childhood [1,2], adolescence [3], and adulthood [4,5]. After preterm birth, the cornea is exposed to a lower temperature than in the intrauterine environment, which may result in less corneal flattening [6]. While preterm babies frequently have a low birth weight due to immaturity at birth, there are also newborns born at term with abnormally low or high birth weight. However, it is not clear whether low or high birth weight as surrogate markers for prenatal growth restriction and fetal supernutrition affect corneal development or whether corneal alterations are only caused by preterm delivery.

Infants born small (SGA) and large for gestational age (LGA) are defined as having abnormal birth weight in relation to gestational age (GA). Moderate LGA is defined as a birth weight larger than the 90th percentile and severe LGA as larger than the 97th percentile, while moderate SGA is defined as a birth weight less than the 10th percentile and severe SGA as less than the 3rd percentile [7]. During pregnancy, weight is estimated by sonographic parameters and monitored closely to assess appropriate fetal growth. Barker and colleagues hypothesized that fetal malnutrition during fetal organ development leads to life-long sequelae in different organ systems and increases the risk for diseases in later life [8]. Overall, more than 30 million infants are born SGA worldwide [9], and the proportion of infants with high birth weight is still dramatically increasing because of increased maternal obesity and diabetes [7].

Recent analyses of preterm children have reported an inverse correlation between birth weight and corneal thickness [10,11]. The Wiesbaden Prematurity Study found that prematurity and low birth weight are associated with increased corneal aberrations in childhood [12], while the Gutenberg Health Study showed that low birth weight (<2500 g) is associated with decreased corneal thickness and increased corneal aberrations in adults aged 40 to 80 years [4]. However, preterm individuals were included in these studies, so the effects of prematurity and prenatal abnormal growth could not be determined separately. This is of clinical importance as corneal thinning may predispose affected persons to different eye diseases, such as keratoconus, and increased corneal aberrations are associated with decreased optical image quality [13]. Alterations in corneal shape can cause deteriorated visual function and low optical image quality [13] and can be evaluated by Scheimpflug imaging [14,15].

Hence, this study evaluated the long-term effects of being born severely or moderately SGA or LGA in adults born at term with respect to corneal aberrations, corneal thickness, and keratoconus indices.

## 2. Materials and Methods

### 2.1. Study Population

The present investigation is part of the Gutenberg Prematurity Eye Study (GPES), a single-center retrospective cohort study at the University Medical Center of the Johannes Gutenberg University Mainz in Germany (UMCM). The GPES involved a prospective ophthalmologic examination in adulthood and recruited individuals (i) born preterm or at term between 1969 and 2002 and (ii) between 18 and 52 years of age at study enrolment.

All study participants were born at the UMCM between 1969 and 2002 with a gestational age ≥37 weeks. For each calendar month (from 1969 to 2002), six randomly selected full-term subjects (3 males and 3 females) with a birth weight between the 10th and 90th percentile were invited and examined. The control group (group 3) of subjects born AGA (10th–90th percentile) in the present study was identical to the control group of the Gutenberg Prematurity Eye Study. Furthermore, for the present study, former term newborns of the UMCM were invited by age matching according to their birth weight percentile (40 participants in each birth weight percentile category group) and classified into severe SGA (BW < 3rd percentile; group 1, *n* = 40), moderate SGA (BW between 3rd and <10th percentile; group 2, *n* = 40), AGA (BW between 10th and 90th percentile; group 3, *n* = 140), moderate LGA (BW between >90th and –97th percentile; group 4, *n* = 40), and severe LGA (BW >97th percentile; group 5, *n* = 40). Scheimpflug imaging of the participants was conducted between 2019 and 2021. In addition, all participants completed a questionnaire, and their relevant medical birth records were reviewed.

All participants provided written informed consent, and this study complied with Good Clinical Practice (GCP), Good Epidemiological Practice (GEP), and the ethical principles of the Declaration of Helsinki. The study protocol and study documents were approved by the local ethics committee of the Medical Chamber of Rhineland–Palatinate, Germany (reference no. 2019-14161; original vote: 29 May 2019, latest update: 2 April 2020).

### 2.2. Assessment of Pre-, Peri-, and Postnatal History

Medical birth records were assessed, including pre-, peri-, and postnatal history (gestational age in weeks, birth weight in kg, gestational diabetes, maternal smoking, preeclampsia, placental insufficiency, and breastfeeding).

### 2.3. Scheimpflug Imaging

Imaging of the anterior eye was performed with a Pentacam HR rotating Scheimpflug camera. To avoid examiner-dependent variance, all measurements were conducted according to standardized operating procedures. The Scheimpflug camera captures 25 images of the anterior segment within two seconds using an optical zone of 6 mm. A refractive index of 1.3375 was used to calculate the measurements of the anterior surface. The following corneal aberrations of the anterior and posterior surface of the cornea were recorded: oblique and vertical astigmatism (Z_2_^−2^ and Z_2_^2^), vertical and horizontal coma (Z_3_^−1^ and Z_3_^1^), vertical and horizontal trefoil (Z_3_^−3^ and Z_3_^3^), spherical aberration (Z_4_^0^), and root mean square of higher-order aberrations (RMS HOA; 3rd up to 8th order) and lower-order aberrations (RMS LOA). Lower-order aberrations included 1st and 2nd order aberrations (tilt, astigmatism, and defocus). Corneal aberrations were computed for the total cornea as well as for the front and back of the corneal surface. In the case of decentration or low quality, the Scheimpflug measurement of that participant was excluded. A board-certified ophthalmologist reviewed each scan. Furthermore, the graphical distribution of corneal parameters was analyzed and skewness was calculated, with approximate normal distribution being assumed for skewness ≤ |1|. Signs of keratoconus were assessed by two board-certified ophthalmologists.

The following parameters were assessed: index of surface variance; index of vertical asymmetry; central keratoconus index; index of height asymmetry; index of height decentration; central corneal thickness in the pupil center; corneal thickness at the apex; corneal center (thinnest corneal thickness) (D0); and circles around the thinnest corneal position with 2 (D2), 4 (D4), 6 (D6), 8 (D8), and 10 (D10) mm diameter. Distance-corrected visual acuity and objective refraction were evaluated in all participants (ARK-1s, NIDEK, Oculus, Wetzlar, Germany).

### 2.4. Covariables

The covariables that may have affected the main outcome measures included sex (female), age (years), gestational age (weeks), birth weight (kg), birth weight percentile, placental insufficiency (yes), preeclampsia (yes), and breastfeeding (yes).

### 2.5. Inclusion/Exclusion Criteria

Only participants with valid corneal tomography were included in the present analysis. Participants with measurements being judged as invalid or of low quality were excluded as were those with a history of corneal refractive, cataract surgery, or ocular trauma as this may have contributed to altered corneal shape.

### 2.6. Statistical Analysis

The primary outcomes were total corneal RMS HOA and LOA as well as central corneal thickness. The secondary outcomes were oblique and vertical astigmatism (Z_2_^−2^ and Z_2_^2^), corneal vertical and horizontal coma (Z_3_^−1^ and Z_3_^1^), corneal vertical and horizontal trefoil (Z_3_^−3^ and Z_3_^3^), corneal spherical aberrations (Z_4_^0^), different keratoconus indices, and corneal thickness in circles around the cornea minimum.

The descriptive statistics stratified by clinical group were computed for the main outcome measures. Absolute and relative frequencies were calculated for dichotomous parameters. Mean and standard deviation were calculated for approximately normally distributed data or median and interquartile range otherwise. Normally distributed ocular parameters of the different groups were compared with ANOVA and Kruskal–Wallis test if they were not normally distributed, and global *p*-values were calculated. Linear regression analysis with generalized estimating equations (GEE) was used to assess associations and to account for correlations between both eyes of one subject. In model #1, univariable analysis of the main outcome measures and GA (weeks), birth weight (kg), birth weight percentile, placental insufficiency (yes), preeclampsia (yes), and breastfeeding (yes) were computed. In model #2, associations with age (years), sex (female), GA (weeks), birth weight percentile (categories), placental insufficiency (yes), preeclampsia (yes), and breastfeeding (yes) were assessed in a multivariable model. Additionally, Spearman’s rank correlation was used to assess the correlation of total corneal aberrations and visual acuity and spherical equivalent. An additional sensitivity analysis was performed with the exclusion of participants wearing contact lenses. As this was an explorative study, a significance level was not defined, and no correction for multiple testing was carried out. Thus, the *p*-values are reported for descriptive purposes and should be interpreted with caution; findings with a *p*-value of <0.05 are discussed as potential differences. Calculations were performed using commercial statistical software (IBM SPSS 20.0; SPSS, Inc., Chicago, IL, USA).

## 3. Results

### 3.1. Participant Characteristics

This study involved 448 eyes of 261 individuals born at term (aged 29.9 ± 9.5 years, 140 females), including 29 subjects with BW <3rd percentile, 32 with BW between 3rd and <10th percentile, 132 with BW between 10th and 90th percentile, 35 with BW between >90th and 97th percentile, and 33 with BW >97th percentile. The participant characteristics are shown in Table 1. Overall, 39 participants were excluded because of invalid Scheimpflug measurement, history of corneal or cataract surgery, or corneal trauma.

### 3.2. Corneal Aberrations

There were no differences between groups in corneal aberrations of the total cornea as well as of the corneal front, except for RMS HOA of the corneal front (*p* = 0.032) (Table 2 and Table S1). There were differences in corneal aberrations of the corneal back for total RMS (*p* = 0.022) and RMS LOA (*p* = 0.024) (Table S2).

Spearman’s rank correlation revealed an association between total corneal aberrations and distant corrected visual acuity (r = 0.11; *p* = 0.023) as well as spherical equivalent (r = −0.17; *p* < 0.001).

### 3.3. Corneal Thickness and Keratoconus Indices

There were no differences in the corneal thickness measured in eight different positions as well as keratoconus indices between the study groups (Table 3 and Table 4).

### 3.4. Uni- and Multivariable Analyses

**Figure 1:**
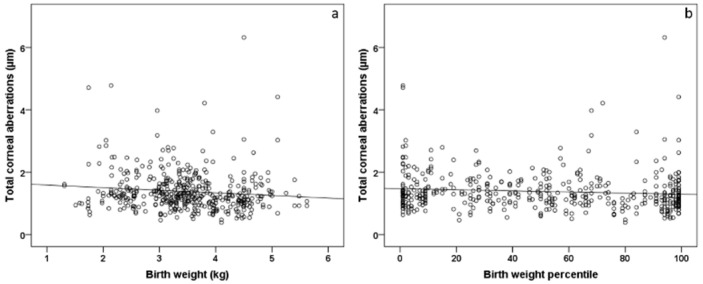
Scatterplot of total corneal aberrations and (**a**) birth weight and (**b**) birth weight percentile in individuals born at term (*n* = 261). Participants with a lower birth weight and birth weight percentile showed increased corneal aberrations.

The univariable analyses revealed an association between central corneal thickness and birth weight (B = 5.386 [95% CI: 1.727; 9.046] µm; *p* = 0.004) and birth weight percentile (B = 0.090 [95% CI: 0.007; 0.172] µm; *p* = 0.034). Furthermore, birth weight and birth weight percentile were associated with higher-order aberrations in the univariable model (Figure 1, Table 5). In the multivariable model, there was still an association between higher-order aberrations and lower birth weight percentile (B = −0.000 [95% CI: −0.001; −0.000] µm; *p* = 0.004) but no associations between selected perinatal factors for lower-order aberrations (Table 5).

### 3.5. Sensitivity Analyses

The results of the association analyses were similar in the sensitivity analysis with the exclusion of participants wearing contact lenses.

## 4. Discussion

This study provides new data about the effects of low and excessive growth on corneal shape in adulthood, with low fetal growth found to be associated with an increase in higher-order aberrations but not corneal thickness. To date, the literature has focused on the association between prematurity and low birth weight with ocular geometry and corneal shape. This study broadens the current knowledge by investigating corneal shape properties in adults born small, appropriate, or large for gestational age at term. The clinical significance of the present findings is the correlation between total corneal aberrations and lower visual acuity.

Prematurity with low birth weight is a known risk factor for corneal changes. The Wiesbaden Prematurity Study (WPS) investigated 226 children born preterm and 259 children born full term at the age of 4 to 10 years. The results showed that preterm children had increased higher-order aberrations irrespective of the postnatal presence of ROP [12]. In addition, Ecsedy et al. compared 50 eyes of 27 children born preterm to 68 eyes of 34 children born full-term aged 7 to 14 years and observed increased higher-order aberrations in the preterm group [16]. A recent report of the Gutenberg Health Study showed that low birth weight (<2500 g) was associated with increased higher- and lower-order aberrations as well as increased spherical aberrations in adults aged 40 to 80 years [17]. Furthermore, these authors observed a thinning of the central corneal thickness in individuals with low birth weight [18]. This data is in line with the results of the Gutenberg Prematurity Eye Study, which analyzed corneal aberrations of 675 eyes of 388 preterm and full-term individuals (aged 28.1 +/− 8.4 years) and found that low birth weight and postnatal ROP treatment were decisive factors for increased higher- and lower-order aberrations. However, it is still unclear whether these effects are also present in term newborns with lower birth weight percentile as surrogate parameters for malnutrition [19]. 

The pathophysiological origins leading to these changes are still unclear, but there is evidence that corneal shape alterations persist until adulthood [20]. While Fiedler et al. postulated that the temperature difference between intra- and extrauterine environment after preterm birth may cause less flattening of the corneal curvature [6], low fetal growth could also play an important role in corneal shape development of preterm and term individuals. The present study supports the latter hypothesis as adults born at term (small, appropriate, or large for gestational age at term) showed changes in higher-order aberrations of the corneal surface. We hypothesize that abnormal fetal nutrition leads to structural alterations of the cornea until adulthood irrespective of prematurity.

Some other parameters have been described in the literature as having an impact on higher-order aberrations. Hashemi et al. evaluated higher-order aberrations in an adult population that included 904 eyes of 577 people and reported an association between higher-order aberrations and older age and shorter axial length, with fewer higher-order aberrations observed in emmetropes and the most higher-order aberrations observed in hyperopes [21]. As there were no significant deviations in the axial length of the different study groups, it is assumed that axial length does not impact the results; rather, nongenetic factors influence corneal shape development, and other parameters are associated with corneal development, such as intraocular pressure [22], lid movements, narrower palpebral position and aperture, and narrower palpebral angle [23,24,25].

In contrast to corneal aberrations, there were no associations between keratoconus indices and corneal thickness in subjects born at term. Our data are in contrast to the investigation of Pan et al. [26], which reported a 19.35 µm thinner central corneal thickness in individuals with low birth weight compared to normal birth weight participants, while the combined effect of birth weight and gestational age on cornea thickness was not statistically significant [20]. The Gutenberg Health Study reported a thinner cornea in participants with low birth weight (<2500 g) compared to the normal birth weight group (2500–4000 g) [4,18]. However, both studies did not exclude participants born preterm, so the authors were unable to separate the effects of prematurity and fetal malnutrition. Thus, our study reveals new data that corneal thickness and keratoconus indices are not affected by the birth weight percentile at term, indicating that prematurity affects corneal thickness.

Overall, corneal aberrations are an important factor for retinal image quality, and some authors have speculated that they are a decisive factor in the process of emmetropization [27], although it is assumed that the effects of corneal aberrations on visual perception are small in relation to myopia and astigmatism [28]. The Gutenberg Prematurity Eye Study recently reported a correlation between corneal aberrations with refractive error and visual acuity. The present study extends these previous findings, showing that the increase in corneal aberrations may be important for visual development and emmetropization processes irrespective of prematurity in individuals born at term. Overall, it remains unclear whether these changes are of subclinical importance, but the correlation between increased corneal aberrations and decreased visual acuity and increased refractive error indicates clinical importance.

### Strengths and Limitations

The present study has several limitations. It is a single-center study and the optical quality of the eye, such as ocular wavefront analysis, was not investigated, so it was not possible to determine whether the increase in corneal higher-order aberrations leads to decreased image quality. Furthermore, the exclusion of participants with previous corneal refractive surgery can lead to a study bias, although none of the participants had previous corneal transplantation or corneal cross-linking. Nonetheless, the strength of the study is the inclusion of only subjects born at term stratified according to birth weight percentiles to investigate the effect of low and high birth weight in a relatively homogeneous group according to gestational age.

## 5. Conclusions

The present study shows that altered fetal growth is associated with changes in higher- and lower-order aberrations of the cornea, indicating that corneal development is influenced by fetal growth irrespective of prematurity, leading to life-long alterations of ocular shape and potentially affecting visual function and refractive error.

## Figures and Tables

**Table 1:** Characteristics of the study sample (*n* = 261) of individuals born at term stratified by birth weight.

	Severe SGA	Moderate SGA	AGA	Moderate LGA	Severe LGA
BW Percentile	<3	3 to <10	10–90	>90 to 97	>97
Number of participants/eyes	29/51	32/54	132/229	35/60	33/54
Sex (Women) (%)	17 (59%)	18 (56%)	77 (58%)	14 (40%)	14 (42%)
Age (y)	29.8 ± 9.1	28.9 ± 9.8	29.8 ± 8.9	30.1 ± 9.8	31 ± 9.8
Birth weight (g)	2105 ± 301	2689 ± 347	3434 ± 388	4306 ± 256	4787 ± 318
Gestational age (wks)	37.9 ± 1.2	38.5 ± 1.4	39.4 ± 1.3	40.0 ± 1.3	40.3 ± 1.1
(min–max)	(37–41)	(37–42)	(37–43)	(37–43)	(37–43)
Preeclampsia (yes)	7 (24%)	1 (3%)	11 (8%)	2 (6%)	6 (18%)
Placental insufficiency (yes)	7 (24%)	0 (0%)	2 (2%)	0 (0%)	0 (0%)
Maternal smoking (yes)	3 (10%)	0 (0%)	6 (5%)	0 (0%)	1 (3%)
Gestational diabetes (yes)	1 (3%)	0 (0%)	1 (1%)	1 (3%)	2 (6%)
Breastfeeding (yes)	12 (41%)	15 (47%)	73 (55%)	22 (63%)	25 (76%)
**Ocular parameters**					
Visual acuity (logMAR) OD	0.0 (0.0/0.0)	0.0 (0.0/0.0)	0.0 (0.0/0.0)	0.0 (0.0/0.0)	0.0 (0.0/0.0)
Visual acuity (logMAR) OD	0.0 (0.0/0.0)	0.0 (0.0/0.0)	0.0 (0.0/0.0)	0.0 (0.0/0.0)	0.0 (0.0/0.0)
Spherical equivalent (diopter) OD	−1.1 ± 2.2	−1.2 ± 1.9	−1.1 ± 2.3	−1.2 ± 2.1	−3.5 ± 1.7
Spherical equivalent (diopter) OS	−1.4 ± 2.0	−1.9 ± 2.5	−0.9 ± 2.1	−1.2 ± 2.1	−0.5 ± 1.4
Axial length (mm) OD	23.2 ± 1.2	23.45 ± 1.0	23.8 ± 1.3	24.3 ± 1.2	23.8 ± 0.8
Axial length (mm) OS	23.3 ± 1.2	23.7 ± 1.2	23.7 ± 1.2	24.1 ± 1.1	23.8 ± 0.9
Intraocular pressure (mmHg) OD	15.4 ± 2.3	15.4 ± 1.8	15.3 ± 2.9	15.1 ± 2.6	14.6 ± 2.8
Intraocular pressure (mmHg) OS	16 ± 2.6	15.5 ± 2.3	15.1 ± 2.9	15.8 ± 3.6	14.8 ± 3.4

g—gram, mm—millimeter, dpt—diopter, y—years, wks—weeks, SGA—small for gestational age, AGA—appropriate for gestational age, LGA—large for gestational age, BW—birth weight, OD—right eye, OS—left eye. Mean ± standard deviation or median and interquartile range.

**Table 2:** Total corneal aberration parameters of the study sample (*n* = 261) grouped according to birth weight percentile.

	Severe SGA	Moderate SGA	AGA	Moderate LGA	Severe LGA	
BW Percentile	<3	3 to <10	10–90	>90 to 97	>97	*p*-Value
Number of participants/eyes	29/51	32/54	132/229	35/60	33/54	
**TOTAL CORNEA**						
**Astigmatism**						
Oblique (Z_2_^−2^ ) OD	0.13 ± 0.49	0.04 ± 0.46	−0.03 ± 0.55	0.05 ± 0.32	0.17 ± 0.41	0.4
Oblique (Z_2_^−2^ ) OS	−0.60 ± 0.32	−0.25 ± 0.65	0.01 ± 0.41	−0.06 ± 0.64	−0.10 ± 0.23	0.4
Vertical (Z_2_^2^) OD	−0.57 ± 0.81	−0.62 ± −0.47	−0.71 ± 0.65	−0.63 ± 0.66	−0.36 ± 0.61	0.7
Vertical (Z_2_^2^) OS	−0.59 ± 0.44	−0.47 ± 1.12	−0.68 ± 0.69	−0.67 ± 0.91	−0.39 ± 0.71	0.9
**Coma**						
Horizontal (Z_3_^1^) OD	0.08 ± 0.19	0.01 ± 0.16	0.01 ± 0.17	0.04 ± 0.20	−0.4 ± 0.15	0.4
Horizontal (Z_3_^1^) OS	−0.02 ± 0.15	−0.04 ± 0.14	0.02 ± 0.17	−0.20 ± 0.18	−0.02 ± 0.14	0.4
Vertical (Z_3_^−1^) OD	−0.04 ± 0.24	−0.03 ± 0.20	0.00 ± 0.21	0.04 ± 0.14	−0.11 ± 0.19	0.5
Vertical (Z_3_^−1^) OS	0.04 ± 0.17	0.10 ± 0.43	0.00 ± 0.21	0.03 ± 0.20	0.02 ± 0.22	0.5
**Trefoil**						
Horizontal (Z_3_^3^) OD	−0.01 ± 0.17	0.00 ± 0.13	0.00 ± 0.13	0.01 ± 0.14	0.02 ± 0.09	0.2
Horizontal (Z_3_^3^) OS	0.00 ± 0.09	0.05 ± 0.58	0.00 ± 0.12	0.00 ± 0.15	0.00 ± 0.16	0.5
Vertical (Z_3_^−3^) OD	−0.09 ± 0.11	−0.07 ± 0.13	−0.08 ± 0.12	−0.11 ± 0.10	−0.06 ± 0.12	0.6
Vertical (Z_3_^−3^) OS	−0.12 ± 0.16	0.05 ± 0.58	−0.05 ± 0.14	−0.06 ± 0.15	−0.04 ± 0.18	0.7
**Spherical aberration** (Z_4_^0^) OD	0.22 ± 0.10	0.23 ± 0.10	0.19 ± 0.09	0.19 ± 0.08	0.19 ± 0.09	0.6
**Spherical aberration** (Z_4_^0^) OS	0.22 ± 0.09	0.26 ± 0.19	0.21 ± 0.12	0.20 ± 0.09	0.26 ± 0.10	0.7
**Corneal aberrations (RMS)**						
Total OD	1.60 ± 0.88	1.26 ± 0.35	1.41 ± 0.59	1.28 ± 1.04	1.39 ± 0.74	0.8
Total OS	1.56 ± 0.87	1.29 ± 0.38	1.39 ± 0.54	1.28 ± 0.59	1.31 ± 0.49	0.5
Higher-order aberrations (HOA) OD	0.40 ± 0.13	0.37 ± 0.07	0.37 ± 0.11	0.36 ± 0.21	0.38 ± 0.12	0.063
Higher-order aberrations (HOA) OS	0.38 ± 0.08	0.40 ± 0.10	0.39 ± 0.11	0.34 ± 0.11	0.37 ± 0.09	0.2
Lower-order aberrations (LOA) OD	1.54 ± 0.89	1.20 ± 0.37	1.35 ± 0.59	1.23 ± 1.02	1.33 ± 0.75	0.8
Lower-order aberrations (LOA) OS	1.50 ± 0.88	1.22 ± 0.38	1.33 ± 0.55	1.23 ± 0.59	1.25 ± 0.50	0.6

Z—Zernicke, OD—right eye, OS—left eye, SGA—small for gestational age, LGA—large for gestational age, AGA—appropriate for gestational age, OD—right eye, OS—left eye, RMS—root mean square.

**Table 3:** Corneal thickness in different locations for each study group.

	Severe SGA	Moderate SGA	AGA	Moderate LGA	Severe LGA	*p*-Value
BW Percentile	<3	3 to <10	10–90	>90 to 97	>97	
Corneal thickness (µm)						
Apex OD	554.1 ± 21.0	549.0 ± 33.2	548.7 ± 34.9	557.9 ± 32.9	559.7 ± 39.3	0.5
Apex OS	558.5 ± 23.3	552.3 ± 30.0	550.2 ± 36.7	564.0 ± 38.8	562.7 ± 30.3	0.2
Pupil OD	553.1 ± 21.2	548.2 ± 33.4	547.5 ± 35.0	557.1 ± 32.5	558.2 ± 39.3	0.5
Pupil OS	557.3 ± 23.5	551.1 ± 30.6	548.5 ± 36.6	561.6 ± 38.3	560.1 ± 30.6	0.2
Corneal thickness in circles around the cornea minimum (µm)						
D 0 mm OD	549.6 ± 22.0	544.6 ± 34.1	545.0 ± 31.9	554.2 ± 32.8	554.9 ± 39.5	0.3
D 0 mm OS	553.0 ± 23.7	546.0 ± 31.3	544.9 ± 33.2	557.0 ± 39.0	554.9 ± 30.8	0.2
D 2 mm OD	559.7 ± 21.6	554.6 ± 33.9	554.8 ± 31.9	563.1 ± 32.7	563.8 ± 39.5	0.4
D 2 mm OS	562.9 ± 23.4	556.3 ± 31.1	554.9 ± 33.2	566.7 ± 39.2	564.4 ± 30.9	0.2
D 4 mm OD	589.4 ± 21.6	585.6 ± 34.6	584.1 ± 32.4	590.5 ± 33.8	591.6 ± 39.5	0.5
D 4 mm OS	592.7 ± 23.9	586.7 ± 32.0	584.3 ± 33.4	594.5 ± 39.9	591.8 ± 31.5	0.2
D 6 mm OD	639.4 ± 22.3	636.3 ± 38.5	634.0 ± 34.8	636.6 ± 36.4	638.5 ± 40.7	0.7
D 6 mm OS	643.1 ± 26.1	637.9 ± 35.8	633.7 ± 35.2	640.3 ± 41.9	637.5 ± 34.4	0.4
D 8 mm OD	718.0 ± 24.4	715.0 ± 46.5	709.9 ± 39.0	707.0 ± 40.4	708.3 ± 44.7	0.7
D 8 mm OS	724.4 ± 28.6	719.0 ± 41.4	709.9 ± 39.1	713.1 ± 45.0	708.6 ± 39.3	0.4
D 10 mm OD	825.3 ± 43.0	825.9 ± 58.6	814.9 ± 49.9	807.9 ± 49.0	803.1 ± 57.3	0.7
D 10 mm OD	838.0 ± 42.6	832.9 ± 50.0	813.0 ± 48.4	821.6 ± 48.8	809.0 ± 50.4	0.1

µm—micrometer, OD—right eye, OS—left eye, SGA—small for gestational age, LGA—large for gestational age, AGA—appropriate for gestational age.

**Table 4:** Keratoconus indices of each study group.

	Severe SGA	Moderate SGA	AGA	Moderate LGA	Severe LGA	
BW Percentile	<3	3 to <10	10–90	>90 to 97	>97	*p*-Value
Number of participants/eyes	29/51	32/54	132/229	35/60	33/54	
Keratoconus (yes)	0 (0%)	0 (0%)	0 (0%)	1 (3.2%)	0 (0%)	
Index of surface variance (ISV)	17.69 ± 7.82	16.54 ± 4.62	16.74 ± 4.48	16.15 ± 6.01	17.28 ± 6.31	0.5
index of vertical asymmetry (IVA)	0.11 ± 0.05	0.12 ± 0.04	0.12 ± 0.05	0.12 ± 0.08	0.13 ± 0.06	0.7
Keratoconus index	1.02 ± 0.02	1.02 ± 0.02	1.02 ± 0.02	1.02 ± 0.02	1.02 ± 0.03	0.053
Central keratoconus index (CKI)	1.01 ± 0.01	1.01 ± 0.01	1.01 ± 0.01	1.01 ± 0.01	1.01 ± 0.01	0.1
Index of height asymmetry (IHA)	5.54 ± 4.67	4.55 ± 3.28	4.97 ± 3.71	4.44 ± 3.79	5.31 ± 3.77	0.5
Index of height decentration (IHD)	0.01 ± 0.01	0.01 ± 0.01	0.01 ± 0.01	0.01 ± 0.10	0.01 ± 0.01	0.9
Smallest radius (Rmin) (mm)	7.42 ± 0.26	7.51 ± 0.23	7.64 ± 0.28	7.75 ± 0.35	7.76 ± 0.27	<0.001

mm—millimeter, OD—right eye, OS—left eye, SGA—small for gestational age, LGA—large for gestational age, AGA—appropriate for gestational age.

**Table 5:** Linear associations of total corneal aberrations with different perinatal parameters (*n* = 261) of individuals born at term (37–43 gestational weeks).

	Unadjusted ^#^		Multivariable ^##^	
	B [95% CI]	*p*	B [95% CI]	*p*
**Central corneal thickness [µm]**				
Gestational age (weeks)	1.741 (−0.327; 3.809)	0.1	0.998 (−1.404; 3.399)	0.4
Birth weight (kg)	5.386 (1.727; 9.046)	0.004	*	*
Birth weight percentile	0.090 (0.007; 0.172)	0.034	0.045 (−0.051; 0.142)	0.4
Placental insufficiency (yes)	−4.766 (−13.830; 4.294)	0.3	−2.992 (−13.701; 7.693)	0.6
Preeclampsia (yes)	0.137 (−9.297; 9.571)	0.9	1.358 (−8.551; 11.266)	0.8
Breastfeeding (yes)	−1.959 (−8.001; 4.083)	0.5	−3.354 (−9.644; 2.936)	0.3
**Higher-order aberration [µm]**				
Gestational age (weeks)	−0.004 (−0.014; 0.007)	0.5	0.001 (−0.010; 0.012)	0.9
Birth weight (kg)	−0.018 (−0.031: −0.004)	0.010	*	*
Birth weight percentile	−0.000 (−0.001; −0.000)	0.005	−0.000 (−0.001; −0.000)	0.004
Placental insufficiency (yes)	0.070 (−0.023; 0.162)	0.1	0.052 (−0.050; 0.155)	0.3
Preeclampsia (yes)	0.029 (−0.004; 0.063)	0.088	0.015 (−0.023; 0.052)	0.4
Breastfeeding (yes)	0.000 (−0.022; 0.022)	0.9	0.013 (−0.006; 0.032)	0.2
**Lower-order aberration [µm]**				
Gestational age (weeks)	−0.008 (−0.065; 0.049)	0.8	0.037 (−0.058; 0.131)	0.5
Birth weight (kg)	−0.081 (−0.166; 0.004)	0.06	*	*
Birth weight percentile	−0.002 (−0.004; 0.000)	0.061	0.001 (−0.005; 0.007)	0.7
Placental insufficiency (yes)	0.070 (−0.457; 0.598)	0.8	−0.050 (−0.626; 0.525)	0.9
Preeclampsia (yes)	−0.026 (−0.173; 0.121)	0.7	−0.075 (−0.240; 0.090)	0.4
Breastfeeding (yes)	−0.027 (−0.148; 0.093)	0.7	0.012 (−0.096; 0.120)	0.8

B—beta, CI—confidence interval, mm—millimeter. In model #1, univariable analysis of the main outcome measures and GA (weeks), birth weight (kg), birth weight percentile, placental insufficiency (yes), preeclampsia (yes), and breastfeeding (yes) were computed. In model ##2, associations with age (years), sex (female), GA (weeks), birth weight percentile (categories), placental insufficiency (yes), preeclampsia (yes), and breastfeeding (yes) were assessed in a multivariable model. * Birth weight was not included in the multivariable model because of the correlation between birth weight and gestational age.

## Data Availability

A.F. had full access to all the study data and has responsibility for the integrity of the data and the accuracy of the data analysis. Statistical analyses were performed by A.F. The analysis presents clinical data and constitutes a major scientific effort with high methodological standards and detailed guidelines for analysis and publication to ensure scientific analyses are of the highest level. Therefore, data are not made available for the scientific community outside the established and controlled workflows and algorithms. To meet the requirements of verification and reproducibility of scientific findings, we offer access to the local database upon request at any time. Interested researchers should make their requests to the coordinating PI (Achim Fieß; achim.fiess@unimedizin-mainz.de). More detailed contact information is available at the UM homepage (www.unimedizin-mainz.de).

## Supplementary Materials

The following supporting information can be downloaded at: https://www.mdpi.com/article/10.3390/jcm11236903/s1, Table S1: Corneal aberrations of the corneal surface of the study sample (*n* = 261) for each study group.; Table S2: Corneal aberrations of the corneal posterior surface of the study sample (*n* = 261) for each study group.

## Abbreviations

BW	birth weight
GA	gestational age
GCP	Good Clinical Practice
GEE	general estimating equations
GEP	Good Epidemiological Practice
UMCM	University Medical Center of the Johannes Gutenberg University Mainz in Germany
