# Supplementary material for: Corneal Aberrations and Thickness in Adults Born Small, Appropriate, or Large for Gestational Age at Term
Source: J Clin Med. 2022 Nov 23;11(23):6903. doi: 10.3390/jcm11236903 (PMC9740638; doi:10.3390/jcm11236903)
Supplement: Supplementary file 1 [file jcm-11-06903-s001.zip › jcm-1998702-supplementary.pdf]

Supplementary Table S1. Corneal aberrations of the corneal surface of the study sample (n=261) for each study group.

BW percentile	Severe SGA<br>< 3	Moderate SGA<br>3 to < 10	AGA<br>10 – 90	Moderate<br>> 90 to 97	Severe LGA<br>> 97	p-value
Number of participants/eyes	29 / 51	32 / 54	132 / 229	35 / 60	33 / 54	
**CORNEAL FRONT**						
**Astigmatism**						
Oblique (Z_2^{-2}) OD	0.17 ± 0.53	0.02 ± 0.43	-0.04 ± 0.57	0.06 ± 0.34	0.15 ± 0.42	0.4
Oblique (Z_2^{-2}) OS	-0.06 ± 0.33	-0.15 ± 0.49	0.04 ± 0.44	-0.02 ± 0.65	-0.09 ± 0.38	0.5
Vertical (Z_2^{2}) OD	-0.72 ± 0.81	-0.83 ± 0.45	-0.87 ± 0.66	-0.78 ± 0.68	-0.51 ± 0.64	0.8
Vertical (Z_2^{2}) OS	-0.74 ± 0.48	-0.61 ± 1.01	-0.83 ± 0.71	-0.83 ± 0.93	-0.55 ± 0.73	0.9
**Coma**						
Horizontal (Z_3^{1}) OD	0.05 ± 0.16	0.01 ± 0.17	0.01 ± 0.17	0.03 ± 0.19	-0.05 ± 0.14	0.4
Horizontal (Z_3^{1}) OS	-0.01 ± 0.15	-0.04 ± 0.18	0.02 ± 0.17	-0.01 ± 0.18	-0.01 ± 0.15	0.3
Vertical (Z_3^{-1}) OD	0.01 ± 0.20	0.00 ± 0.18	0.02 ± 0.21	0.06 ± 0.14	-0.09 ± 0.19	0.4
Vertical (Z_3^{-1}) OS	0.00 ± 0.18	0.08 ± 0.30	0.02 ± 0.21	0.05 ± 0.20	0.04 ± 0.23	0.6
**Trefoil**						
Horizontal (Z_3^{3}) OD	0.01 ± 0.09	0.00 ± 0.08	-0.01 ± 0.10	0.00 ± 0.12	0.03 ± 0.08	0.3
Horizontal (Z_3^{3}) OS	0.00 ± 0.09	0.17 ± 0.60	0.00 ± 0.11	0.01 ± 0.14	0.01 ± 0.12	0.2
Vertical (Z_3^{-3}) OD	-0.06 ± 0.09	-0.03 ± 0.13	-0.05 ± 0.11	-0.7 ± 0.08	-0.04 ± 0.09	0.9
Vertical (Z_3^{-3}) OS	-0.08 ± 0.14	-0.09 ± 0.35	-0.03 ± 0.11	-0.05 ± 0.12	-0.06 ± 0.16	0.6
**Spherical aberration** (Z_4^{0}) OD	0.26 ± 0.08	0.27 ± 0.10	0.24 ± 0.08	0.24 ± 0.8	0.24 ±0.08	0.6
**Spherical aberration** (Z_4^{0}) OS	0.26 ± 0.09	0.29 ± 0.16	0.25 ± 0.12	0.24 ± 0.08	0.29 ± 0.10	0.8
**Corneal aberrations (RMS)**						
Total OD	1.81 ± 0.86	1.51 ± 0.36	1.61 ± 0.57	1.53 ± 1.19	1.56 ± 0.78	0.7
Total OS	1.82 ± 0.92	1.53 ± 0.30	1.59 ± 0.52	1.52 ± 0.63	1.49 ± 0.54	0.5
Higher-order aberrations	0.41 ± 0.11	0.38 ± 0.06	0.38 ± 0.10	0.37 ± 0.25	0.37 ± 0.09	0.032
Higher-order aberrations	0.39 ± 0.09	0.41 ± 0.09	0.39 ± 0.09	0.35 ± 0.12	0.36 ± 0.09	0.069
Lower-order aberrations	1.76 ± 0.87	1.46 ± 0.37	1.56 ± 0.57	1.48 ± 1.16	1.51 ± 0.78	0.8
Lower-order aberrations	1.78 ± 0.92	1.48 ± 0.30	1.54 ± 0.52	1.48 ± 0.62	1.44 ± 0.55	0.5

All aberrations are reported in µm. Z—Zernicke, OD—right eye, OS—left eye, SGA—small for gestational age, LGA—large for gestational age, AGA—appropriate for gestational age, RMS - root mean square.

Supplementary Table S2. Corneal aberrations of the corneal posterior surface of the study sample (n=261) for each study group.

BW percentile	Severe SGA < 3	Moderate SGA 3 to < 10	AGA 10 – 90	Moderate > 90 to 97	Severe LGA > 97	p-value
Number of participants /	29 / 51	32 / 54	132 / 229	35 / 60	33 / 54	
<u>CORNEAL BACK</u>						
**Astigmatism**						
Oblique (Z_2^{-2}) OD	-0.02 ± 0.28	0.02 ± 0.1	0.02 ± 0.12	-0.01 ± 0.09	0.01 ± 0.09	0.3
Oblique (Z_2^{-2}) OS	0.01 ± 0.10	0.00 ± 0.12	-0.03 ± 0.13	-0.03 ± 0.15	0.00 ± 0.12	0.8
Vertical (Z_2^{2}) OD	0.24 ± 0.15	0.32 ± 0.13	0.28 ± 0.15	0.26 ± 0.14	0.22 ± 0.12	0.9
Vertical (Z_2^{2}) OS	0.26 ± 0.14	0.24 ± 0.17	0.28 ± 0.15	0.27 ± 0.22	0.24 ± 0.18	0.9
**Coma**						
Horizontal (Z_3^{1}) OD	0.03 ± 0.08	0.00 ± 0.06	0.00 ± 0.03	0.01 ± 0.03	0.02 ± 0.03	0.9
Horizontal (Z_3^{1}) OS	-0.01 ± 0.03	-0.02 ± 0.1	-0.01 ± 0.03	-0.01 ± 0.03	-0.01 ± 0.03	0.1
Vertical (Z_3^{-1}) OD	-0.06 ± 0.09	-0.05 ± 0.06	-0.03 ± 0.05	-0.04 ± 0.04	-0.02 ± 0.04	0.086
Vertical (Z_3^{-1}) OS	-0.05 ± 0.04	-0.02 ± 0.01	-0.03 ± 0.05	-0.04 ± 0.04	-0.04 ± 0.06	0.5
**Trefoil**						
Horizontal (Z_3^{3}) OD	-0.03 ± 0.19	0.00 ± 0.11	0.01 ± 0.08	0.01 ± 0.05	-0.01 ± 0.06	0.1
Horizontal (Z_3^{3}) OS	0.00 ± 0.05	-0.03 ± 0.03	0.00 ± 0.06	0.00 ± 0.04	-0.02 ± 0.06	0.3
Vertical (Z_3^{-3}) OD	-0.03 ± 0.11	-0.03 ± 0.14	-0.02 ± 0.06	-0.03 ± 0.05	-0.01 ± 0.05	0.2
Vertical (Z_3^{-3}) OS	-0.03 ± 0.06	0.00 ± 0.01	-0.02 ± 0.06	-0.00 ± 0.06	0.03 ± 0.08	0.1
**Spherical aberration** (Z_4^{0}) OD	-0.16 ± 0.05	-0.15 ± -0.04	-0.15 ± 0.03	0.16 ± 0.03	-0.15 ± 0.03	0.9
**Spherical aberration** (Z_4^{0}) OS	-0.16 ± 0.04	-0.15 ± 0.01	-0.15 ± 0.03	0.15 ± 0.03	-0.15 ± 0.03	0.8
**Corneal aberrations (RMS)**						
Total OD	0.83 ± 0.18	0.85 ± 0.17	0.78 ± 0.15	0.73 ± 0.27	0.76 ± 0.20	0.4
Total OS	0.89 ± 0.20	0.85 ± 0.15	0.80 ± 0.16	0.80 ± 0.15	0.76 ± 0.14	0.022
Higher-order aberrations	0.19 ± 0.04	0.19 ± 0.04	0.18 ± 0.03	0.17 ± 0.06	0.17 ± 0.03	0.1
Higher-order aberrations	0.20 ± 0.02	0.20 ± 0.04	0.19 ± 0.04	0.17 ± 0.03	0.18 ± 0.03	0.059
Lower-order aberrations	0.80 ± 0.18	0.83 ± 0.17	0.76 ± 0.15	0.71 ± 0.27	0.74 ± 0.20	0.5
Lower-order aberrations	0.87 ± 0.20	0.83 ± 0.15	0.78 ± 0.16	0.78 ± 0.15	0.74 ± 0.14	0.024

All aberrations are reported in µm. Z—Zernicke, OD—right eye, OS—left eye, SGA—small for gestational age, LGA—large for gestational age, AGA—appropriate for gestational age, RMS - root mean square.
